# Supplementary material for: Reconciling Mining with the Conservation of Cave Biodiversity: A Quantitative Baseline to Help Establish Conservation Priorities
Source: PLoS One. 2016 Dec 20;11(12):e0168348. doi: 10.1371/journal.pone.0168348 (PMC5173368; doi:10.1371/journal.pone.0168348)
Supplement: S1 Dataset — (ZIP) [file pone.0168348.s002.zip › Taxa/Serra Norte/SN_2013/N5S/Lista N5S-16.pdf]

CAVIDADE N5S-0016

Classe	Ordem	Fam/Outros	Gên/Outros	Espécie	S-U	U-S	Zon.
Arachnida	Acari	Rhagidiidae		sp.20	X		E
Arachnida	Araneae	Filistatidae		jovem	X		E
Arachnida	Araneae	Ochyroceratidae	*Ochyrocera*	sp.2	X		E
Arachnida	Araneae	Oonopidae		jovem	X		E
Arachnida	Araneae	Pholcidae		jovem	X		E
Arachnida	Araneae	Prodidomidae	*Lygromma*	sp.1	X		E
Arachnida	Araneae	Salticidae		sp.		X	E
Arachnida	Araneae	Scytodidae	*Scytodes*	sp.1	4		E
Arachnida	Araneae	Tetragnathidae	*Azilia*	sp.		X	E
Arachnida	Araneae	Theridiosomatidae	*Plato*	sp.1	X		E
Arachnida	Opiliones	Minuidae		sp.		X	E
Arachnida	Opiliones	Zalmoxidae		sp.1	X		E
Entognatha	Collembola	Paronellidae		sp.3	X	X	E
Entognatha	Diplura	Campodeidae		sp.1	X		E
Insecta	Archaeognatha	Meinertellidae		sp.1	X		E
Insecta	Blattodea			jovem		X	E
Insecta	Coleoptera	Curculionidae	Scolytinae	sp.2	X		E
Insecta	Heteroptera	Nabidae	*Arachnocoris*	jovem	X		E
Insecta	Homoptera	Cixiidae		jovem	X		E
Insecta	Hymenoptera	Formicidae	*Camponotus*	sp.1	X		E
Insecta	Hymenoptera	Formicidae	*Crematogaster*	sp.1	X		E
Insecta	Hymenoptera	Formicidae	*Odontomachus*	sp.1	X		E
Insecta	Hymenoptera	Formicidae	*Solenopsis*	sp.1	X		E
Insecta	Isoptera	Termitidae	*Nasutitermes*	sp.	X		E
Insecta	Lepidoptera			jovem	X		E
Insecta	Mantodea	Mantidae		jovem	1		E
Insecta	Orthoptera	Gryllidae	Eneopterinae	sp.	1		E
Insecta	Orthoptera	Phalangopsidae	*Paraclodes*	sp.	5		E
Insecta	Psocoptera	Archipsocidae	*Archipsocus*	sp.1	X		E
Insecta	Psocoptera	Archipsocidae	*Archipsocus*	sp.2	X		E
Onychophora	Onychophora	Peripatidae	*Peripatus*	sp.1	1		E
Amphibia	Anura	Strabomantidae	*Pristimantis*	cf. *fenestratus*	2		E
Mammalia	Chiroptera	Emballonuridae	*Peropteryx*	*kappleri*	2		E
